# Phenolic Acids Profiles and Phenolic Concentrations of Emmer Cultivars in Response to Growing Year under Organic Management

**DOI:** 10.3390/foods12071480

**Published:** 2023-03-31

**Authors:** Magdaléna Lacko-Bartošová, Lucia Lacko-Bartošová, Ľubomír Kobida, Amandeep Kaur, Jan Moudrý

**Affiliations:** 1 Institute of Agronomic Sciences, Faculty of Agrobiology and Food Resources, Slovak University of Agriculture in Nitra, Tr. A. Hlinku 2, 949 76 Nitra, Slovakia; 2 Department of Agroecosystems, Faculty of Agriculture and Technology, University of South Bohemia in České Budějovice, Branišovská 1645/31a, 37005 České Budějovice, Czech Republic

**Keywords:** emmer, common wheat, phenolic acids profiles, DPPH, phenolic compounds

## Abstract

Phenolic compounds, especially phenolic acids (PAs), are believed to be one of the major contributors to the antioxidant activity of cereal grains. This study determined and compared phenolic concentration, radical scavenging activities, individual PA concentrations of emmer cultivars, and breeding lines to common wheat in a three-year controlled field experiment under organic management. It was found that common wheat had the highest ability to scavenge DPPH radicals (51.7%), followed by emmer Farvento (35.4%). DPPH scavenging activity of bound phenolic extracts was higher compared to free ones. Total phenolic concentration was the highest for common wheat (1902.6 µg FAE g^−1^ DM) compared to the highest level of all emmer cultivars—Farvento (1668.3 µg FAE g^−1^ DM). The highest PAs concentration was determined for emmer Farvento (431.3 µg g^−1^ DM) and breeding line PN 4-41 (424.5 µg g^−1^ DM). Free PAs concentration was the lowest for common wheat (29.5 µg g^−1^ DM). The dominant free PA was ferulic (66.3%), followed by syringic (11.7%), sinapic (7.4%), *p*-hydroxybenzoic (5.3%), salicylic (3.8%), *p*-coumaric (3.6%), and caffeic (2.1%). Bound ferulic acid accounted for 94.0% of total bound PAs, followed by *p*-coumaric (2.8%), *p*-hydroxybenzoic (0.8%), syringic (0.8%), caffeic (0.6%), sinapic (0.6%), and salicylic (0.4%). Emmer cultivar Farvento was distinguished by the highest concentration of individual free and bound forms of PAs. Effect of growing year was more evident on the concentration of free PAs compared to bound PAs. Extremely dry and hot weather during maturity stages has a negative impact on analysed free and bound PAs.

## 1. Introduction

Emmer wheat (*Triticum dicoccon* Schrank) is one of the earliest domesticated crop species, it was one of the basic plants in Neolitic agriculture and a determining factor for the beginning of agriculture. Emmer was a staple crop over milenia [1]. Wheat evolution studies showed that tetraploid wild emmer wheat (*T. dicoccoides*) containing A^u^A^u^BB genomes (*Triticum urartu* as a pollen donor, genome A^u^A^u^, and probably female parent *Aegilops speltoides* Tausch with BB genome; 2n = 4x = 28) were central to the domestication process. Wild emmer wheat is at the core of wheat domestication evolution [2]. Archaeological findings indicate a polycentric origin of agriculture and multiple-site independent domestication of wild emmer. Emmer wheat has spread mainly across Iran, Eastern Turkey, Ethiopia, Italy, Spain, India, Volga Basin, and mountainous regions of Western Europe [3]. On the basis of eco-morphological description and geographical distribution, Vavilov (1964) and Dorofeev et al. (1979), in [4] distinguished four subspecies: *abyssinicum* Vav. (Abyssinian emmer), *asiaticum* Vav. (Eastern emmer), *dicoccum* (European emmer), and *maroccanum* Flaksb. (Moroccan emmer). Furthermore, they subdivided the subspecies into convarieties, hence, emmer wheat intraspecific classification is quite complex. Its long history of cultivation in a broad range of eco-geographical environments demonstrate high variability for agronomic, morphological, and quality traits.

Phytochemicals are defined as bioactive non-nutrient plant compounds. As a result, they have received increasing attention due to their beneficial effects on human health as well as the protection against oxidative injury in plants under abiotic stresses [5]. These are classified as carotenoids, phenolics, alcaloids, N-containing compounds, and organosulphur compounds. The most studied phytochemicals are phenols and carotenoids [6]. Generally, phenolic compounds are categorized as phenolic acids, flavonoids, stilbens, coumarins, and tannins. Phenolic acids, as predominant phenolics in all cereals, are categorized as derivates of benzoic acid (e.g., gallic, *p*-hydroxybenzoic, vanillic, syringic, protocateuchic acids) and cinnamic acid (e.g., ferulic, *p*-coumaric, caffeic, sinapic acids). Different antioxidant activities are affected by the hydroxylation and methoxylation of the aromatic ring. The predominant mode of the phenolic acids’ antioxidant activity is hydrogen atom donation [7]. It is well known that free radicals cause antioxidation of unsaturated lipids in food. Antioxidants are believed to intercept the free radical chain during oxidation and to donate hydrogen from the phenolic hydroxyl groups, thereby forming a stable end product [8]. Oxygen free radicals have been implicated as causative agents in conditions such as cancer, ischemia, atherosclerosis, Parkinson’s disease, and aging. Free radicals are generated by metabolism and exogenous factors, e.g., carcinogenic compounds and UV light. Although the ability of living cells to neutralize oxidative free radicals is limited, it is thought that extrinsic antioxidants can enhance the protection of essential cellular components and physiological process [9]. The interaction between antioxidant molecules and radicals causes the reduction in DPPH radical absorption induced by antioxidants [10]. Natural antioxidants, largely present in many plants, help to limit cellular and molecular damage and contribute to preventing mutagenesis, carcinogenesis, cardiovascular diseases, and type 2 diabetes, owing to their radical scavenging activities [11]. In recent years, there has been increasing interest in emmer wheat as a source of physiologically active components, such as dietary fibre, protein, vitamins, minerals, and antioxidants [12]. Various studies showed that emmer has remarkable nutritional properties, high protein content, and secondary components that make it useful as an ingredient in functional foods [13,14]. Emmer is suggested in the diets of patients with allergies, colitis, and high blood cholesterol levels [15].

In cereal grains, phenolic acids occur in soluble free, soluble conjugated, and insoluble bound forms. The majority of phenolic acids (95%) are present in the insoluble bound form, linked to cell wall components, e.g., lignin, cellulose, and proteins, often esterified to the arabinose groups of arabinoxylans [16,17]. The quantity of phenolic acids can vary, depending on wheat species, cultivars, but also processing technology. Phenolic acids predominantly occur in wheat bran, aleurone layer, in the parts usually eliminated by milling [18,19]. Because the number of phenolic acids in whole grain and bran varies greatly, the composition in food will be impacted by cereal type and milling procedure [20]. A substantial diversity in phenolic acids and flavonoids has been documented amongst wheat cultivars; hence, selecting wheat cultivars with greater amounts of phenolics would be a viable way to achieve a favourable effect on public health [21]. The antioxidant properties of phenolic acids may aid in the production of cereal-based functional meals [22]. Due to the high concentration of hydroxycinnamic acids, the antioxidant activity of wheat grain fractions is negatively proportional to the aleurone level. Wheat grain’s aleurone layer and pericarp contain 98% of the total ferulic acid [18]. The most abundant phenolic acid in cereals grains is ferulic acid, which accounted for 75% found in the kernel husk, 15% in the endosperm, and the rest in the aleurone layer [16]. Ferulic acid makes up the majority of the PAs found in cereals, although other acids, including *p*-coumaric and vanillic, are also frequently found [23]. The capacity of phenolic acids to bind free radicals declines as follows: gallic, caffeic, benzoic, sinapic, syringic, ferulic, *p*-coumaric, vanillic, and 4-hydroxybenzoic [17]. Across wheat species, the trend for total polyphenols content was einkorn > emmer > common wheat > spelt, while for the total phenolic acids it was einkorn > spelt > emmer > common wheat [24].

There have been published a number of studies of the contents and compositions of phytochemicals in ancient wheat species. In spite of that, comparisons of these species with common and durum wheat are rare. Comparisons are further complicated by different methods used for PAs analysis between the laboratories [25]. Several studies showed that emmer had greater total phenolic acids than einkorn and bread wheat controls [11,26]. Li et al. [21] found that emmer, durum, and bread wheat had the greatest phenolic acids content; however, these values did not differ substantially from spelt and einkorn. Additionally, the higher grain antioxidant activity was observed in emmer than einkorn varieties, which means that emmer can be a promising source of these compounds [27,28]. The activity of DPPH in emmer was substantially lower than in einkorn [29].

In recent years, emmer wheat rich genetic resources are considered a valuable reservoir for wheat improvement. They include many agronomic attributes, such as abiotic stress tolerances (salt, drought, heat), biotic stress resistance/tolerance (rusts, powdery mildew, *Fusarium* head blight), kernel weight and size, yield, micronutrient concentrations, and protein quality, which can enrich cultivated bread and durum wheat to improve the yield stability against changing climate [30,31,32,33]. Hence, the relevant antioxidant activity, along with superior contents of proteins, tocols, carotenoids, and polyphenols, reinforce the potential of emmer as a nutritionally superior cereal source. Despite the relatively high genotypic significance, environmental factors or climatic parameters (such as rainfall and temperature) can influence the phenolic compounds. Water deficit and higher average temperatures during the grain-filling stage increased the synthesis of such components in different cereals [24]. Understanding the impact of genotype, year, and their interaction on selected phenolic compounds could be utilized to improve breeding efforts to create ancient wheat grain rich in specified health components. However, there is less information documented in the literature regarding the influence of the cropping environment on phenolic acid variation.

Thus, the present investigation focused on ancient wheat emmer, and variations across tested cultivars and breeding lines, compared with common wheat. The objectives were to evaluate and compare the concentrations of (a) free, bound, total phenolic compounds, (b) free, bound, total phenolic acids, and (c) to determine antioxidant activity using DPPH assay. Bioactive compounds were assessed in the context of an organic management system and changing weather conditions during three growing seasons.

## 2. Materials and Methods

### 2.1. Plant Material, Sample Preparation

Six winter cultivars and breeding lines of emmer (Agnone, Guardiaregia, Farvento, Molise sel Colli, PN 6-37, and PN 4-41) and one winter bread wheat cultivar (Laudis) were grown under organic farming management at the experimental fields of the Institute of Agronomic Sciences in Nitra, Slovakia (48°19′ N, 18°07′ E), during three consecutive growing periods (2015–2017). Experimental area was located on a Haplic luvisol developed on proluvial sediments mixed with loess. The elevation of experimental fields was 177–178 m. a. s. l., the climate was continental with average long-term (1951–2000) annual temperature 9.9 °C and average annual long term (LT) precipitations of 547.6 mm.

Ancient wheat—emmer and common wheat—were grown after common pea as a pre-crop, no synthetic fertilizers and pesticides were used during vegetation period. Soil cultivation was based on ploughing to the depth of 0.22 m, and during vegetative period mechanical weed regulation treatments were used several times. Plant nutrition was based on residual nitrogen after N—fixing pre-crop (common pea)—and the application of farmyard manure in the amount of 30 t per hectare, three years before emmer and common wheat cultivation. Emmer cultivars were sown between 15th and 21st of October every year. Field experiment was designed as a randomized complete block in four replicates. Plots of ancient wheat—emmer—were hand-harvested at grain humidity below 14% during July (on 16 July 2015, 21 July 2016, and 14 July 2017).

Flowering and maturation periods of experimental years were differentiated. First year (2015) was characterized by very dry June and July (only 15.4% and 29.0% of LT precipitations); this dry period was accompanied by high and extremely high temperatures (in July, 3.6 °C over long-term average). In the second growing year (2016), this period started with wet May, when recorded precipitations represented 163.0% of long-term average, temperature was at the level of LTA. June was warm and very dry (only 21.7% of LT precipitations). July was warmer than LT temperature, about 1.4 °C, and very wet (227.1% of LT precipitations). In the third growing year (2017), deficiencies of precipitations were recorded in May and June. Moreover, in June, the highest temperature (2.9 °C over LT average) was measured out of all experimental years. Precipitations in July achieved 101.2 % of LT average.

Monthly temperature (t) and precipitation (p) data during flowering and maturation periods with calculations of temperature (∆t) and precipitation difference (%) to long-term data are given in Table 1.

After harvest, emmer spikes were dehulled on laboratory dehuller KMPP 300 (JK Machinery, Czech Republic) and stored at 4 °C. Then, emmer samples were hand-cleaned and sieved using analytical sieve shaker (Retsch GmbH, type AS 200, Haan, Germany). The grains smaller than 2.2 mm (remainers) were not taken to further analyses. Whole-grain meal was prepared by milling all samples on FQC—109 laboratory mill (Kapacitív Kft., Budapest, Hungary), using 250 µm sifter. Next, the whole-grain meal was defatted according to Krygier et al. [34] and Pang et al. [22] with a slight modification, as follows: 10 g of sample was transferred to 50 mL ST 50 tube from disintegrator ULTRA-TURRAX Tube Drive Control (IKA, Staufen, Germany), and defatted with hexane at a 2:1 ratio (*v*/*w*) at 1000 rpm two times. For filtration, Whatman No.1 filter paper was used and dried at room temperature.

### 2.2. Extraction Procedure for Free and Bound Phenolics

Free and bound phenolic extracts were prepared by the method of Van Hung et al. [35] with modifications in four replicates. The defatted samples (2 g) were mixed with 15 mL of 80% methanol, using sonification for 15 min. (Bandelin DT 100, Berlin, Germany). The samples were centrifuged at 8965× *g* (9000 rpm) (Hettich Universal 320, Hettich, Tuttlingen, Germany).

After centrifugation, the supernatant was withdrawn and used for determination of free phenolics. The residua were re-extracted twice, and all supernatants were mixed together. All phenolic extracts were concentrated with rotary vacuum evaporator RVO 400 (INGOS, Praha, Czech Republic). The samples were filled up with 60% methanol to a final volume of 10 mL in 50 mL tubes. The samples were kept in a freezer (−20 °C, 4–5 days) until further use.

After extraction of free phenolics, alkaline hydrolysis was used to release bound phenolics from cereal residua. The 15 mL of 4N NaOH was added in 50 mL tube. After sonification for 15 min, the samples were acidified to pH 2 with 6 M HCl. Finally, the bound phenolics were extracted using diethyl ether four times. The pooled diethyl ether extracts were evaporated to dryness and bound phenolics were reconstituted in 10 mL 60% methanol and stored in freezer (−20 °C, 7 days) until use.

### 2.3. HPLC Analysis of Phenolic Acids (PAs)

The quantitative and qualitative analyses of the PAs were performed using HPLC/MS/MS system AGILENT 1260 (AGILENT, Santa Clara, CA, USA) equipped with DAD detector, Triple Quadrupole 6410 MS/MS detector, autosampler, and multicolumn thermostat, as reported by Brandoliny et al. [23]. The analytical column Symmetry© C18 5µm, 4.6 × 250 mm (WATERS, Milford, MA, USA) was used. Mobile phase was of 0.2% formic acid in water (*v*/*v*) (solvent A) and 0.2% formic acid in methanol (*v*/*v*) (solvent B). The gradient program was as follows: 10–26% B 0–5 min, 26–65% B 5–10 min, 65% B 10–25 min, and 65–10% B 25–30 min. Flow rate was 1 mL/min. Injection volume was 40 µL. DAD detector was set at 280 and 320 nm. The mass spectrometer was operated in ESI mode, gas temperature 325 °C, vaporizer 200 °C, gas flow 5 L/min, nebulizer 60 psi, and capillary voltage 2500 V. The phenolic acids were detected by comparison to the retention times of standards and confirmed by the ion mass from MS/MS detector.

The HPLC/MS/MS system was operated under the Mass Hunter software (AGILENT, Santa Clara, CA, USA). For peak quantification, the calibration curves were constructed for sinapic acid, syringic acid, ferulic acid, caffeic acid, salicylic acid, *p*-hydroxybenzoic acid, and *p*-coumaric acid. All standards were in concentration 10, 50, 100, and 200 µg/mL solution (60% MeOH). The calibration curves were linear and on the basis of the calibration curves the detection limits were 0.09, 0.08, 0.94, 0.03, 0.05, and 0.06 µg/mL, respectively. The concentration levels of individual PAs in whole-grain meal were expressed in µg/g on a dry matter basis (DM).

### 2.4. Total Phenolic Concentration Assay

The free and bound phenolics were determined using Fiolin-Ciocalteu’s reagent according to Van Hung et al. [35] with some modification. A mixture containing 150 µL of extract was added to 750 µL of deionized water. Solution was then oxidized by adding 300 µL Fiolin-Ciocalteu’s reagent, incubated in the dark at room temperature for 8 min, and neutralized with 300 µL of 20% sodium carbonate solution (*w*/*v*). Then, samples were left to stand for 120 min in a dark place, centrifuged at 16,060× *g* (13,000 rpm) for 10 min, and the absorbance of clear supernatants was measured at 765 nm using spectrophotometer UV-1800 (SHIMADZU, Kyoto, Japan). A standard calibration solution was prepared using ferulic acid and the content of free and bound phenolics was quantified and expressed as ferulic acid equivalent (FAE) per gram of the dry sample. Analyses were performed in four replicates.

### 2.5. DPPH Radical Scavenging Assay

The free radical scavenging activity was determined by the DPPH assay. The scavenging activity against 2,2-diphenyl-1-picrylhydrazil hydrate (DPPH) radical (95%, Sigma-Aldrich Chemie, Steinheim, Germany) was measured according to the method of Van Hung et al. [35] and Huang et al. [36]. In the presence of an antioxidant, the purple colour of DPPH fades and the change of absorbency can be measured spectrophotometrically. For each measurement, 0.6 mL of sample extract was mixed with 0.9 mL DPPH solution (12.5 mg of DPPH in 100 mL 80% methanol). After the solution reaction for 30 min in a dark place, the absorbance was measured at 515 nm using spectrophotometer UV-1800 (SHIMADZU, Kyoto, Japan). The measurements were performed in four analytical replicates. The radical scavenging activity was calculated by the following formula: % DPPH scavenging = [(AB − AC)/AB] × 100, where AB = absorption of a blank sample at t = 0 min; AC = absorption of cereal sample at t = 30 min. Methanol (0.6 mL) was used as control.

### 2.6. Statistical Analysis

Analysis of variance (ANOVA) was used for statistical evaluation of experimental data. Significant differences between the factors (cultivars, growing years, replicates, interactions) were determined by F-test. Significantly different means were calculated by Fisher’s least significant difference (LSD) test. Pearson’s correlation coefficients were calculated to evaluate the relationships between selected parameters. All statistical analyses were carried out by the STATISTICA software version 13.0 (TIBCO Software Inc., Tulsa, OK, USA).

## 3. Results and Discussion

### 3.1. Radical Scavenging Activity (RSA)

The reduction capability of DPPH is determined by the decrease in its absorbance induced by antioxidants. A compound with significant antioxidant capacity successfully traps the radical in the DPPH radical scavenging method, halting its expansion and subsequent chain reaction [37,38]. In the present study, the radical scavenging activities of the different free and bound phenolic extracts were investigated using the DPPH method. The results obtained showed that all tested cultivars and breeding lines of emmer and common wheat demonstrated antioxidant activity, their RSA varied in a wide range (Table 2), from 26.16 to 51.72% (common wheat) in total DPPH values. According to the ability of phenolic extracts to scavenge free radicals in DPPH reaction systems, there were remarkable differences in the antioxidant properties of emmer and common wheat. Extracts of emmer cultivars were able to scavenge between 26.16% (PN 6-37) and 35.41% (Farvento) of DPPH radicals, and the differences between cultivars and breeding lines of emmer were significant. Along with Farvento, Molise and PN 4-41 also achieved the highest values. The DPPH scavenging activity of free phenolic extracts was also the highest in common wheat (20.58% of inhibition), and all emmer cultivars achieved significantly lower values, in the range from 10.51% (Guardiaregia) to 14.11% (Farvento). DPPH scavenging activity of bound phenolic extracts was higher compared to free phenolic extracts, ranging from 15.44% (PN 6-37) to 31.14% (common wheat). Thus, the extract of common wheat had the highest ability to scavenge DPPH radicals, followed by emmer Farvento (21.31%), and the lowest inhibition was determined for emmer PN 6-37. None of the emmer cultivars achieved the level of common wheat in the ability to scavenge the DPPH radicals. In contrast to our results, Abdel-Aal and Rabalski [39] noted that diploid, tetraploid, and primitive wheat had better DPPH scavenging capacity compared to modern wheat, but with some exceptions. The capacity for scavenging activity against DPPH by wheat antioxidants may vary, depending on the concentration of individual bioactive compounds in extracts and their synergic effects. Sandhu et al. [40] reported DPPH activity of wheat in the range of 13–22%. In our experiment, a significant effect of growing year was found for all free, bound, and total DPPH scavenging activity, with the highest level of RSA in 2016 (40.21%, total DPPH), followed by 2015 and 2017.

### 3.2. Total Phenolic Concentration (TPC)

The phenolic compounds’ secondary metabolites, which are produced by the plants throughout their growth and in response to stress, are good oxygen radical scavengers because they have an electron reduction potential that is lower than that of oxygen radicals. Analysis of variance indicated that the concentrations of free, bound, and total phenolic compounds were significantly affected by all tested cultivars and breeding lines (C) of emmer and common wheat. Statistical analyses also showed the highly significant effect of growing year (Y) and their interaction (C × Y). The highest concentration of total phenolic compounds was determined in common wheat (1902.6 µg FAE g^−1^ DM), while all emmer cultivars achieved lower values ranging from 1286.8 µg FAE g^−1^ DM (PN 6-37) to 1668.3 µg FAE g^−1^ DM (Farvento) (Table 2); however, the differences between emmer cultivars and breeding lines were significant. Results revealed that common wheat and Farvento had the highest and equivalent free phenolic concentration (271.4 µg FAE g^−1^ DM) compared to other emmer cultivars and breeding lines, whereas Agnone had the lowest (209.7 µg FAE g^−1^ DM). The contribution of free phenolics to the total ranged from 14.3% in common wheat up to 15.7% in Agnone. Bound phenolic compounds represented 84.1% of the total phenols with the highest concentration found in common wheat (1631.2 µg FAE g^−1^ DM), whereas all emmer cultivars had lower values ranging from 1071.6 µg FAE g^−1^ DM in PN 6-37 to 1396.9 µg FAE g^−1^ DM in Farvento. Farvento was the dominating one out of all emmer cultivars. Bound phenolics contributed to the total ones in the range from 83.3% (PN 6-37) to 85.5% (common wheat). It was clearly observed that *Triticun aestivum* had the significantly highest bound and total phenolic concentration and Guardiaregia had the significantly lowest. According to Serpen et al. [11], emmer had a higher phenolic concentration than einkorn and bread wheat controls. The opposite trend was observed by Lachman et al. [27], who determined a lower concentration of total phenolic compounds in spring wheat (604 µg GAE g^−1^ DM) compared to emmer (638 µg GAE g^−1^ DM). In our experiment, the highest levels of free, bound, and total phenolic concentration were found in 2016, followed by 2015 and 2017. Our findings are partly consistent with those of Lachman et al. [27], who found significant differences between two growing years in their study of emmer, einkorn, and bread wheat genotypes due to lower rainfall and higher temperatures during cereal ripening stages. In the present study, highly significant and positive correlation was observed between total radical scavenging activity (total DPPH) and total phenolic concentration (r = 0.916) between bound DPPH and bound phenolic concentration (r = 0.92), and furthermore, free DPPH correlated with free phenolics (r = 0.77). This correlation is supported by the findings of other researchers [28,41], who also found a significant positive relationship between total phenolic content and antioxidant activity. These findings imply that phenolic compounds in grains have a direct effect on antioxidant capacity [42]. The established correlation between total phenolic concentration and total antioxidant activity suggests that phenolic compounds are directly responsible for antioxidant action.

### 3.3. Total Phenolic Acids (TPAs)

The total phenolic acids are represented by the sum of free and bound forms of these compounds [43]. Analysis of variance indicated that the concentrations of free, bound, and total phenolic acids were significantly affected by all tested cultivars and breeding lines of emmer and common wheat. Contrary to the findings of total phenolics, the highest concentration of total phenolic acids was observed in emmer Farvento (431.3 µg g^−1^ DM) and PN 4-41 (424.5 µg g^−1^ DM), and the lowest in PN 6-37 and Guardiaregia (Table 3). Concentrations of TPAs for common wheat were within the range of emmer cultivars. Results revealed the lowest free PAs concentration in common wheat (29.50 µg g^−1^ DM), all emmer cultivars and breeding lines achieved significantly higher values in a range from 30.79 µg g^−1^ DM to 37.17 µg g^−1^ DM, with the highest concentration determined for Farvento. The average share of free PAs in total PAs was 8.48%. Concentration of bound PAs was also the highest for emmer Farvento (394.1 µg g^−1^ DM) and breeding line PN 4-41 (389.2 µg g^−1^ DM), with significant differences between tested samples. The share of bound PAs in the total concentration of PAs was high and represented 92.52% on average. Similar results were reported in other research [44,45], when bound forms of PAs accounted for the majority of all PAs, while free PAs were recorded only up to 10% of total PAs. Across emmer cultivars, Farvento had the highest free, bound, and total phenolic acids concentration. Similarly to our results, the highest content of total phenolic acids was observed in emmer compared to common wheat [21,24]. Our experiment found a significant effect of growing year for free, bound, and total phenolic acids, with the highest level in 2016 followed by 2015 and 2017. Significant interaction of cultivars with growing year indicated different responses of cultivars to meteorological conditions. The influence of the growing year on phenolic acids concentration has been scantily studied. In addition, Stracke et al. [46] found that the impact of growing year was the most significant factor in their three-year investigations under organic farming conditions. However, several studies showed that minimum rainfall throughout the heading and maturity stages of wheat was related with an increase in phenolic content [23]. Climate, farming techniques, and underlying genes all influence plant metabolic pathways, and there is a substantial relationship between the concentration of bioactive components and environmental parameters such as precipitation and temperature [25].

### 3.4. Individual Phenolic Acids

In the present study, individual phenolic acids concentrations were analysed for free and bound fractions. Phenolic acids included ferulic, *p*-hydroxybenzoic (*p*-HBA), *p*-coumaric, syringic, sinapic, salicylic, and caffeic (Table 4, Table 5 and Table S1). As expected, the predominant phenolic acid in all cultivars was ferulic, which accounted for 67.10% of free PAs in common wheat and from 65.76% (Guardiaregia) to 66.02% (Farvento) in emmer. The second predominant PA was syringic acid, with an average 11.67% share on total free PAs, followed by sinapic acid (7.39%), *p*-HBA (5.34%), salicylic (3.77%), *p*-coumaric acid (3.59%), and, last, caffeic acid (2.12%). The significantly lowest concentrations of all analysed free PAs (except ferulic) were found in common wheat, the concentration of free ferulic acid (19.87 µg g^−1^ DM for common wheat) was at the level of emmer PN 6-37 (20.32 µg g^−1^ DM), which was the lowest of all emmer cultivars.

Cultivar Farvento was characterised by the highest concentration of all seven analysed free phenolic acids, and at the same time the emmer breeding line PN 6-37 achieved the lowest concentration of all analysed PAs out of all emmer samples. Comparable with the results of total free PAs (Table 3), the second highest concentrations of all analysed individual PAs were found in breeding line PN 4-41. Good bioavailability of free ferulic acid was reported by Anson et al. [47]. Its absorption occurred mostly from the small intestine, therefore for phenolic antioxidant intake, free extractable polyphenols are especially important. Non-extractable polyphenols can be used in the diet after acidic hydrolysis [48,49]. From this point of view, the significantly higher concentrations of free phenolic acids in all emmer samples compared to common wheat may provide health benefits in the human diet.

The concentrations of all individual free Pas were significantly affected by the growing year. The highest concentrations of all Pas were noted in 2016, followed by 2017 and 2015. In 2015, significantly lowest concentrations of all free Pas were determined, except sinapic acid with no significant difference between 2017 and 2015. The maturation period of the 2015 growing year was extremely dry and very warm, and from June until harvest only 17.2 mm of rainfall was recorded. The last growing year, 2017, was also characterized by extremely dry May and June, and from June until harvest 60.2 mm of rainfall was measured, but June was the warmest during the whole experiment. The maturation period of the 2016 growing year had the most favourable meteorological conditions for synthesis of phenolic acids, where a wet May was followed by a very dry and warm June. July was 1.4 °C warmer than LTA, but no deficiency of rainfall was recorded (from June until harvest there was 92.0 mm of rainfall). The impact of growing year on total variation was high and represented 81.2% (sinapic acid) to 89.0% (ferulic acid). The share of cultivars on total variation was much lower and accounted for 8.6% (ferulic acid) to 13.4% (syringic acid).

There were also significant interactions found between cultivars and growing years, for ferulic and *p*-HBA at the level of *p* < 0.001, and for *p*-coumaric and caffeic at *p* < 0.05. The significantly highest concentrations across all individual Pas and all cultivars and breeding lines were found in 2016. Concentrations of free ferulic and free *p*-HBA were not significantly different in 2015 and 2017, only for PN 6-37 and cultivar Agnone. Other cultivars and breeding lines achieved significant differences in concentrations of these two acids during all experimental years.

For concentrations of free *p*-coumaric and caffeic acids, the interaction between cultivar and growing year showed that Farvento had significant differences in caffeic acid concentrations during all years of the experiment. The same pattern was found for free *p*-coumaric acid, only for cultivar Guardiaregia. All other cultivars and breeding lines did not show any differences in the 2015 and 2017 growing years in the concentration of these two phenolic acids.

Dominance of ferulic acid among all phenolic acids in wheat species, accounting for more than 72% of free PAs and over 95% of bound forms, was reported by Baranski et al. [50]. In contrast to our results, a lower proportion of free phenolic acids in emmer than in *Triticum aestivum* was noted by Li et al. [21]. The scarcity of data on the presence of free phenolic acids in ancient wheat has been declared, and at the same time more in-depth research employing a variety of ancient and modern wheat species and cultivars has been advocated for [25].

The bound phenolic acids are the components found in larger concentrations in cereals, and these compounds are closely associated with the seed’s fibrous components, such as lignins, cellulose, arabinoxylans, and other indigestible polysaccharides, which restrict their release in the small intestine [43,48]. The bound forms of PAs represented the majority of all PAs in the cereals under study. Results showed that when all cultivars and breeding lines were analysed together, that bound ferulic acid accounted for 94.02% of the total bound PAs, followed by *p*-coumaric acid (2.77%), *p*-HBA (0.85%), syringic acid (0.76%), and caffeic (0.65%), whereas sinapic (0.56%) and salicylic acid (0.38%) had the lowest concentrations. Emmer cultivar Farvento was distinguished from all the other cultivars by the highest concentration of all individual bound Pas, except syringic acid. For breeding line PN 4-41, the highest concentrations of ferulic acid, *p*-coumaric, salicylic, and caffeic acid were also measured, so the total concentration of bound PAs was at the level of the Farvento cultivar. All the other cultivars of emmer were lower in the quantity of all analysed individual phenolic acids. Our findings showed that the highest concentration of syringic acid was observed in common wheat; furthermore, in this species, *p*-HBA and salicylic acids were also detected within the highest levels. However, the binding of ferulic acid to polysaccharides in the cereal matrix limits its bioavailability and the insoluble (bound) fraction did not show cellular antioxidant activity [51]. In this study, the caffeic acid concentration of common wheat was one of the lowest (2.14 µg g^−1^ DM), and significant differences among other cultivars and breeding lines were detected. On the contrary, Baranski et al. [50] reported that only caffeic acid quantity was not different among wheat species of einkorn, emmer, and spelt. The concentrations of all bound individual phenolic acids were found to be significantly affected by the growing year; moreover, highly significant interactions among cultivars (breeding lines) and growing years were determined for all bound phenolic acids. The highest concentrations of all bound phenolic acids were observed in 2016, as it was the result of free individual phenolic acids. However, the concentrations of the analysed bound Pas were not significantly different in 2015 and 2017, except syringic and *p*-HBA acids, with the lowest values in 2015. The influence of growing year on total variation was more evident for bound Pas compared to free ones, when the share of growing year was in the range from 95.5% (*p*-coumaric acid) to 91.9% (caffeic acid), while the share of cultivars was from 3.1% (*p*-coumaric acid) to 4.9% (syringic acid). The effect of environmental factors (rainfall, air temperature) was investigated in several studies, but the results were inconsistent. Negative correlation was observed between high temperature and total phenolic concentration [52,53], while Alexieva et al. [54] found an increase in soluble phenols values in wheat subjected to drought and UV-B stressors.

It Is worth noting that for cultivars Farvento, Guardiaregia, and breeding line PN 6-37, significant differences in the quantity of ferulic, *p*-HBA, and syringic acids were observed across all experimental years; moreover, Farvento and Guardiaregia also showed substantial differences during all three years in concentrations of caffeic and salicylic acids. Significant differences in all experimental years were noticed for common wheat in terms of the quantity of ferulic, *p*-HBA, and sinapic acids. Other cultivars (Agnone, Molisse, and PN 4-41) responded differently to the environmental conditions of growing years and the differences in concentrations of phenolic acids in 2015 and 2017 were not significant. Baranski et al. [49] reported for ancient wheat species more frequent effect of cultivation year on free PAs (except for syringic acid) quantity, compared to bound PAs, for which the effect of year was found to be important for *p*-HBA, *p*-coumaric, salicylic, and syringic acids. Bound caffeic acid concentration was not different among wheat species. Climate, meteorological conditions, agricultural systems, agri-technological measures, such as crop rotation and tillage, but also genotypes can possibly affect the metabolic pathways in crops and the concentration of secondary metabolites differently, depending upon the crop species [24]. Until now, no comprehensive literature review or meta-analysis have been presented on this topic, therefore no definitive conclusions have been drawn yet [50].

## 4. Conclusions

The presented results indicated a high antioxidant potential and health benefits of evaluated emmer cultivars and breeding lines, as an important source of phenolic acids in the human diet. We conclude that there is a significant effect of emmer cultivars, breeding lines, growing year, and their interactions on phenolic concentrations, radical scavenging activity, and phenolic acids profiles in both free and bound forms. Emmer cultivars (breeding lines) with significant concentrations of phenolic compounds, antioxidant activity, and phenolic acids profiles were identified. Results suggested that these compounds are highly dependent on genotypes. The outcomes of the study demonstrated that the effect of growing year was more evident on the concentration of free phenolic acids compared to bound phenolic acids. Warmer weather without precipitation deficiency during the ripening period was related to the increase in PAs concentrations. The effect of extremely dry and hot weather during the maturity stages was negative. The understanding of bioactive compounds compositions could provide valuable information for breeding efforts to produce varieties rich in health-promoting compounds or support the cultivation and use of this type of cereals.

## Figures and Tables

**Table 1:** Meteorological data during maturation periods.

Year	Month	t (°C)	∆t (°C)	p (mm)	LTA (%)
Long-term average (1951–2000)	May	15.2		56.0	
	June	18.3		66.2	
	July	20.0		59.3	
2015	May	15.1	−0.1	69.5	124.1
	June	19.9	1.6	10.2	15.4
	July	23.6	3.6	17.2	29.0
2016	May	15.0	−0.2	91.3	163.0
	June	20.3	2.0	14.4	21.7
	July	21.4	1.4	134.7	227.1
2017	May	16.6	1.4	14.0	25.0
	June	21.2	2.9	26.1	39.4
	July	21.7	1.7	60.0	101.2

LTA—long-term average.

**Table 2:** Effect of wheat cultivars and growing years on phenolic concentrations (µg FAE g^−1^ DM) and DPPH (%) in whole-grain meal.

Species, Cultivars, Breeding Lines	Phenolic Compounds (μg FAE g^−1^ DM)			DPPH (%)		
	Free	Bound	Total	Free	Bound	Total
*T. aestivum*, Laudis	271.4 ± 46.4 a	1631.2 ± 258.5 a	1902.6 ± 303.6 a	20.58 ± 4.03 a	31.14 ± 6.15 a	51.72 ± 10.05 a
*T. dicoccon*						
Agnone	209.7 ± 36.3 e	1123.3 ± 187.0 d	1332.0 ± 221.4 d	11.37 ± 1.59 d	17.16 ± 2.24 de	28.53 ± 3.78 d
Guardiaregia	220.2 ± 37.0 d	1108.3 ± 174.8 de	1328.5 ± 210.7 de	10.51 ± 1.69 e	16.68 ± 3.30 e	27.18 ± 4.89 e
Molise	237.6 ± 42.0 c	1244.7 ± 215.0 c	1482.3 ± 254.5 c	11.89 ± 2.26 c	17.92 ± 3.37 cd	29.81 ± 5.57 c
Farvento	271.4 ± 45.7 a	1396.9 ± 242.4 b	1668.3 ± 286.0 b	14.11 ± 2.38 b	21.31 ± 3.35 b	35.41 ± 5.67 b
PN 6-37	215.2 ± 37.9 de	1071.6 ± 167.8 e	1286.8 ± 201.9 e	10.72 ± 2.11 e	15.44 ± 2.66 f	26.16 ± 4.61 f
PN 4-41	249.2 ± 37.7 b	1271.1 ± 205.8 c	1520.3 ± 240.9 c	12.09 ± 1.71 c	18.15 ± 3.70 c	30.24 ± 5.39 c
*p* cultivars (C)	***	***	***	***	***	***
2015	221.6 ± 24.5 b	1155.4 ± 182.2 b	1377.0 ± 201.6 b	12.16 ± 2.81 b	17.94 ± 4.50 b	30.10 ± 7.26 b
2016	290.8 ± 32.2 a	1533.9 ± 232.0 a	1824.6 ± 260.5 a	15.92 ± 4.39 a	24.29 ± 6.62 a	40.21 ± 10.90 a
2017	205.3 ± 23.8 c	1102.4 ± 166.0 c	1307.7 ± 185.2 c	11.03 ± 2.95 c	16.83 ± 4.32 c	27.86 ± 7.24 c
*p* year (Y)	***	***	***	***	***	***
*p* C × Y	**	**	***	***	***	***

All values are means ± standard deviations (SD) for four replicates and three years of experiment (n = 12 for cultivars, n = 28 for years). Values in columns (among cultivars and years) followed by the same letter are not significantly different at *p* < 0.05. F-test from ANOVA significant at *** *p* < 0.001, ** *p* < 0.01.

**Table 3:** Effect of wheat cultivars and growing years on the sum of analysed phenolic acids (PAs) concentration (µg g^−1^ DM) in whole-grain meal.

Species, Cultivars, Breeding Lines	Free PAs	Bound PAs	Total PAs
*T. aestivum*, Laudis	29.50 ± 5.82 e	366.5 ± 67.1 b	396.0 ± 72.3 b
*T. dicoccon*			
Agnone	33.44 ± 4.49 c	369.1 ± 90.5 b	402.5 ± 94.8 b
Guardiaregia	32.89 ± 4.06 c	331.1 ± 65.0 d	363.9 ± 69.0 d
Farvento	37.17 ± 4.23 a	394.1 ± 73.7 a	431.3 ± 77.8 a
Molise	32.78 ± 5.36 c	350.0 ± 102.2 c	382.8 ± 107.3 c
PN-6-37	30.79 ± 2.77 d	330.3 ± 62.6 d	361.1 ± 65.2 d
PN-4-41	35.28 ± 4.57 b	389.2 ± 107.9 a	424.5 ± 112.1 a
*p* cultivars (C)	***	***	***
2015	29.62 ± 2.23 c	305.6 ± 30.4 b	335.2 ± 30.8 b
2016	38.92 ± 2.73 a	468.0 ± 45.7 a	506.9 ± 47.5 a
2017	30.83 ± 3.39 b	310.8 ± 27.7 b	341.7 ± 29.5 b
*p* year (Y)	***	***	***
*p* C × Y	***	***	***

All values are means ± standard deviations (SD) for four replicates and three years of experiment (n = 12 for cultivars, n = 28 for years). Values in columns (among cultivars and years) followed by the same letter are not significantly different at *p* < 0.05. F-test from ANOVA significant at *** *p* < 0.001.

**Table 4:** Effect of wheat cultivars and growing years on free individual phenolic acids concentration (µg g^−1^ DM) in whole-grain meal.

Species, Cultivars, Breeding Lines	Ferulic Acid	*p*-HBA ^1^	*p*-Coumaric Acid	Syringic Acid	Sinapic Acid	Salycylic Acid	Caffeic Acid
*T. aestivum*, Laudis	19.87 ± 4.25 d	1.53 ± 0.29 e	1.05 ± 0.21 e	3.30 ± 0.58 d	2.08 ± 0.37 e	1.06 ± 0.22 e	0.61 ± 0.15 e
*T. dicoccon*							
Agnone	22.00 ± 2.99 c	1.80 ± 0.25 c	1.21 ± 0.16 c	3.98 ± 0.50 b	2.46 ± 0.35 c	1.27 ± 0.18 c	0.71 ± 0.10 bc
Guardiaregia	21.63 ± 2.64 c	1.79 ± 0.25 c	1.19 ± 0.16 c	3.84 ± 0.46 b	2.46 ± 0.34 c	1.26 ± 0.16 c	0.70 ± 0.08 c
Farvento	24.54 ± 2.83 a	1.99 ± 0.25 a	1.33 ± 0.16 a	4.31 ± 0.46 a	2.77 ± 0.34 a	1.41 ± 0.16 a	0.80 ± 0.09 a
Molise	21.61 ± 3.48 c	1.75 ± 0.31 c	1.18 ± 0.19 c	3.83 ± 0.67 b	2.44 ± 0.40 c	1.26 ± 0.23 c	0.70 ± 0.11 c
PN-6-37	20.32 ± 1.86 d	1.64 ± 0.13 d	1.10 ± 0.10 d	3.62 ± 0.33 c	2.29 ± 0.21 d	1.16 ± 0.11 d	0.66 ± 0.07 d
PN-4-41	23.24 ± 3.04 b	1.87 ± 0.26 b	1.27 ± 0.16 b	4.18 ± 0.54 a	2.63 ± 0.37 b	1.35 ± 0.17 b	0.74 ± 0.09 b
*p* cultivars (C)	***	***	***	***	***	***	***
2015	19.54 ± 1.43 c	1.58 ± 0.13 c	1.06 ± 0.09 c	3.49 ± 0.30 c	2.20 ± 0.19 b	1.11 ± 0.12 c	0.63 ± 0.06 c
2016	25.78 ± 1.79 a	2.08 ± 0.19 a	1.39 ± 0.11 a	4.49 ± 0.42 a	2.87 ± 0.29 a	1.47 ± 0.13 a	0.82 ± 0.06 a
2017	20.35 ± 2.22 b	1.65 ± 0.20 b	1.11 ± 0.12 b	3.61 ± 0.43 b	2.27 ± 0.27 b	1.18 ± 0.14 b	0.66 ± 0.08 b
*p* year (Y)	***	***	***	***	***	***	***
*p* C × Y	***	***	*	**	**	**	*

^1^ *p*-hydroxybenzoic acid. All values are means ± standard deviations (SD) for four replicates and three years of experiment (n = 12 for cultivars, n = 28 for years). Values in columns (among cultivars and years) followed by the same letter are not significantly different at *p* < 0.05. F-test from ANOVA significant at *** *p* < 0.001, ** *p* < 0.01, and * *p* < 0.05.

**Table 5:** Effect of wheat cultivars and growing years on bound individual phenolic acids concentrations (µg g^−1^ DM) in whole-grain meal.

Species, Cultivars, Breeding Lines	Ferulic Acid	*p*-HBA ^1^	*p*-Coumaric Acid	Syringic Acid	Sinapic Acid	Salycylic Acid	Caffeic Acid
*T. aestivum*, Laudis	345.2 ± 63.4 b	3.33 ± 0.40 a	9.36 ± 2.39 cd	3.10 ± 0.72 a	2.01 ± 0.33 cd	1.44 ± 0.20 a	2.14 ± 0.47 c
*T. dicoccon*							
Agnone	346.9 ± 85.0 b	3.09 ± 0.77 b	10.36 ± 2.69 b	2.76 ± 0.65 c	2.08 ± 0.51 bc	1.37 ± 0.33 b	2.43 ± 0.60 b
Guardiaregia	311.2 ± 61.1 d	2.82 ± 0.55 c	9.33 ± 1.99 cd	2.46 ± 0.43 e	1.83 ± 0.34 e	1.24 ± 0.25 c	2.19 ± 0.41 c
Farvento	370.5 ± 69.1 a	3.34 ± 0.79 a	11.00 ± 2.19 a	2.89 ± 0.51 b	2.20 ± 0.38 a	1.50 ± 0.27 a	2.60 ± 0.50 a
Molise	328.9 ± 96.2 c	2.93 ± 0.90 c	9.87 ± 2.79 bc	2.60 ± 0.69 d	1.97 ± 0.58 d	1.34 ± 0.39 b	2.34 ± 0.69 b
PN-6-37	310.5 ± 58.8 d	2.83 ± 0.61 c	9.23 ± 1.84 d	2.48 ± 0.48 e	1.86 ± 0.39 e	1.22 ± 0.22 c	2.15 ± 0.39 c
PN-4-41	365.9 ± 101.4 a	3.12 ± 0.94 b	10.91 ± 3.11 a	2.94 ± 0.82 b	2.18 ± 0.60 ab	1.45 ± 0.43 a	2.58 ± 0.70 a
*p* cultivars (C)	***	***	***	***	***	***	***
2015	287.4 ± 29.0 b	2.58 ± 0.35 c	8.34 ± 0.78 b	2.30 ± 0.22 c	1.74 ± 0.21 b	1.16 ± 0.14 b	2.04 ± 0.23 b
2016	439.9 ± 43.1 a	3.98 ± 0.41 a	13.18 ± 1.33 a	3.55 ± 0.42 a	2.58 ± 0.30 a	1.76 ± 0.19 a	3.01 ± 0.39 a
2017	292.4 ± 26.2 b	2.67 ± 0.28 b	8.51 ± 0.91 b	2.39 ± 0.24 b	1.73 ± 0.16 b	1.19 ± 0.11 b	1.99 ± 0.26 b
*p* year (Y)	***	***	***	***	***	***	***
*p* C × Y	***	***	***	***	***	***	***

^1^ *p*-hydroxybenzoic acid. All values are means ± standard deviations (SD) for four replicates and three years of experiment (n = 12 for cultivars, n = 28 for years). Values in columns (among cultivars and years) followed by the same letter are not significantly different at *p* < 0.05. F-test from ANOVA significant at *** *p* < 0.001.

## Data Availability

All data generated or analysed in this study are included in this published article and its Supplementary Information files.

## Supplementary Materials

The following supporting information can be downloaded at https://www.mdpi.com/article/10.3390/foods12071480/s1, Table S1: The set of data used for the evaluation of the effect of species (emmer and common wheat), cultivars, growing year on phenolic compounds, phenolic acids, and DPPH.
